# Environmental Arsenic Exposure and Microbiota in Induced Sputum

**DOI:** 10.3390/ijerph110202299

**Published:** 2014-02-21

**Authors:** Allison G. White, George S. Watts, Zhenqiang Lu, Maria M. Meza-Montenegro, Eric A. Lutz, Philip Harber, Jefferey L. Burgess

**Affiliations:** 1 Mel and Enid Zuckerman College of Public Health, University of Arizona, Tucson 85724, USA; E-Mails: gyson@email.arizona.edu (A.G.W.); ealutz@email.arizona.edu (E.A.L.); pharber@email.arizona.edu (P.H); 2 Department of Pharmacology and University of Arizona Cancer Center, Tucson, AZ 85724, USA; E-Mail: gwatts@email.arizona.edu; 3 Statistical Consulting Laboratory, University of Arizona, Tucson, AZ 85712, USA; E-Mail: zlu@arizona.edu; 4 Department of Biotechnology and Food Sciences, Instituto Technologico de Sonora, Sonora 85000, Mexico; E-Mail: mmeza@itson.edu.mx

**Keywords:** arsenic, microbiota, sputum

## Abstract

Arsenic exposure from drinking water is associated with adverse respiratory outcomes, but it is unknown whether arsenic affects pulmonary microbiota. This exploratory study assessed the effect of exposure to arsenic in drinking water on bacterial diversity in the respiratory tract of non-smokers. Induced sputum was collected from 10 subjects with moderate mean household water arsenic concentration (21.1 ± 6.4 ppb) and 10 subjects with low household water arsenic (2.4 ± 0.8 ppb). To assess microbiota in sputum, the V6 hypervariable region amplicons of bacterial 16s rRNA genes were sequenced using the Ion Torrent Personal Genome Machine. Microbial community differences between arsenic exposure groups were evaluated using QIIME and Metastats. A total of 3,920,441 sequence reads, ranging from 37,935 to 508,787 per sample for 316 chips after QIIME quality filtering, were taxonomically classified into 142 individual genera and five phyla. *Firmicutes* (22%), *Proteobacteria* (17%) and *Bacteriodetes* (12%) were the main phyla in all samples, with *Neisseriaceae* (15%), *Prevotellaceae* (12%) and *Veillonellacea* (7%) being most common at the genus level. Some genera, including *Gemella*, *Lactobacillales*, *Streptococcus*, *Neisseria* and *Pasteurellaceae* were elevated in the moderate arsenic exposure group, while *Rothia*, *Prevotella*, *Prevotellaceae Fusobacterium* and *Neisseriaceae* were decreased, although none of these differences was statistically significant. Future studies with more participants and a greater range of arsenic exposure are needed to further elucidate the effects of drinking water arsenic consumption on respiratory microbiota.

## 1. Introduction

Arsenic is a metalloid element commonly found in drinking water, and globally its levels are above 10 ppb for an estimated 160 million people or approximately 2% of the human population [1]. The concentration of arsenic in drinking water in the United States (U.S.) averages 2 ppb [1], although some geographic hot spots have much higher levels. Since 2001, the U.S. Environmental Protection Agency (EPA) has set the arsenic Maximum Contaminant Level (MCL) at 10 ppb [2]. 

Exposure to arsenic is a major worldwide health concern because of its toxicity [3] and carcinogenicity [4]. In addition to its known effects on multiple organ systems [1] and its role as a lung, skin, and bladder carcinogen [4], studies have implicated ingestion of high concentrations of arsenic in drinking water as a cause of bronchiectasis [5] and immune system suppression [6]. In addition, there is evidence that ingestion of arsenic-contaminated water leads to reduced lung function [7] and non-malignant lung disease [8,9]. 

The pulmonary microbiota (microbiome) reflects the totality of bacteria in the lungs. Recent culture-independent techniques demonstrate that diverse microbial communities reside in the human respiratory tract [10,11] and their composition or characterization may be altered by disease status [11,12,13,14,15], or exposure [16]. For example, there is a significant disparity between the microbiome in the airway of healthy individuals and those with chronic obstructive pulmonary disease (COPD), with the former having have more diverse bacterial population than the latter [11,15]. The same holds true for exposure to smoking [16]. Evidence of relationships between non-commensal bacterial species detected in the respiratory tract and asthma development has also been reported [17]. 

We have previously demonstrated that arsenic exposure in drinking water affects the level of proteins within the lung and inhibits epithelial cell wound repair [18,19,20]. We therefore hypothesized that arsenic exposure through drinking water would also alter resident bacterial flora of the lungs. The objective of this exploratory study was to compare the airway microbiome of subjects with moderate drinking water arsenic concentrations to those with low exposure, including evaluation of the effect of arsenic exposure on microbial community diversity.

## 2. Experimental Section

### 2.1. Ethics Statement

The original Binational Arsenic Exposure Study (BAsES) was approved by the University of Arizona Institutional Review Board [21]. The current study was reviewed and considered exempt based on retrospective analysis limited to existing biologic specimens and clinical data without personal identifiers. 

### 2.2. Study Area and Selection of Subjects

BAsES evaluated subjects between 2006 and 2007 for arsenic exposure in southern Arizona and northern Mexico; details of the study design and methods have been published [21]. For the current study, 20 archived sputum samples were evaluated from non-smokers over 18 years of age from the area surrounding Ciudad Obregon, Sonora, Mexico [22], who relied on community wells for water and had lived for many years in their respective communities. The twenty samples were randomly selected, including ten samples being from subjects with moderately high mean household water arsenic concentrations (13.8 to 28.8 ppb), and 10 subjects with low household water arsenic (<3.5 ppb). 

### 2.3. Prior Sample Collection and DNA Extraction

During the BAsES study, water samples were collected from all available sources of water reported as consumed in and outside the home. First morning urine voids were collected. Water and urine samples were analyzed for total arsenic by the Southwest Hazardous Waste Program, Hazard Identification Core at The University of Arizona. In addition, As III, As V, methylarsonic acid (MMA V), and dimetylarsinic acid (DMAV) were analyzed in the urine samples [21]. The sum of species was defined as the sum of As III, As V, methylarsonic acid (MMAV) and dimetylarsinic acid (DMA V). Sputum was then induced by having subjects inhale sterile 3% saline aerosol (Baxter, Deerfield, IL, USA) from DeVilbiss Ultra-Neb 99HD ultrasonic nebulizers (DeVilbiss Healthcare, Somerset, PA, USA) set on maximum output [23]. Subjects were then encouraged to cough up sputum every 2 min for a total period of 30 min. To reduce salivary contamination, subjects discarded any mouth-held saliva before each coughing episode. After collection, the sputum samples were suspended with an equal volume of 10% Sputolysin (Calbiochem, San Diego, CA, USA) and incubated at room temperature for 15 min. The samples were then centrifuged at 1,900 rpm for 15 min to separate the cell pellet, which was removed and stored at −80 °C.

For the current study, the frozen cell pellets were thawed on ice, suspended with lysozyme (final concentration 2.9 mg/mL, Sigma-Aldrich Corp. (St. Louis, MO, USA) and incubated for 60 min at 37 °C, with vortexing every 20 minutes. Genomic DNA was extracted using the QIAamp DNA Minikit (Qiagen, Valencia, CA, USA) using the manufacturer’s protocol. DNA was eluted with 100 µL buffer AE (Qiagen) and stored at −20 °C. As a negative control, the same procedure was used with sterile TE buffer with no PCR products detected, indicating lack of contamination in the reagents used. The concentration of reconstituted genomic DNA was determined with the Quant-iT OliGreen quantification kit according to the manufacturer’s instructions using a TBS-380 mini-fluorometer (Turner BioSystems, Sunnyvale, CA, USA).

### 2.4. PGM Sequencing

The V6 (*E. coli* reference nucleotides 969–1073) hypervariable region of the bacterial 16s rRNA gene was sequenced on an Ion Torrent PGM machine (Life Technologies, Grand Island, NY, USA) at the Southwest Environmental Health Science Center Genomics Facility Core. V6 amplicons were generated by PCR using 40 nanograms of template DNA and custom primers consisting of PGM-specific sequencing adaptor, a bar code sequence and 16s sequence (forward primer 16s sequence targeting E. coli reference sequence 1054–1073: ACGAGCTGACGACARCCATG, reverse primer 16s sequence targeting E. coli reference sequence 969–984 ACGCGARGAACCTTACC) under the following conditions: PCR was done using the Phusion High-Fidelity Polymerase (Thermo Scientific, Waltham, MA, USA) and the supplied 5× GC buffer and amplified with the following condition, 98 °C for 1 min than 30 cycles of 98 °C for 10 seconds, 66.8 °C for 30 seconds, than 72 °C for 30 seconds. PCR amplicons were gel purified after electrophoresis on 1% agarose gel. The band of interest was excised and placed onto a small bed of silicanized glass wool packed into a 0.65 mL tube with a pin hole at the bottom. The band was spun at 14,000 rpm for 20 min and the eluate was captured in a 1.5 mL microfuge tube. The PCR product was then purified using Agencourt AMPure XP beads (Beckman Coulter, Indianapolis, IN, USA) according to manufacturer’s instruction. DNA was then quantified using the Qubit DNA High Sensitivity Assay (Life Technologies) and diluted to 64 pM. Next, 5 µL was seeded into a Ion OneTouch Template reaction following the user guide catalog number 4468660 Rev E (Life Technologies), after which template beads were enriched using the Ion OneTouch ES system. Enriched beads were quantitated using the Qubit Ion Sphere Quality Control kit. Sequencing libraries were loaded on Ion Torrent 314 chips and sequenced according to the manufacturer’s protocol using the IonPGM 200 Sequencing Reagents Kit following (Publication # 4474246 Rev G). FASTQ [24] files were processed using cutadapt version 1.2.1 [19.5] to remove primer sequence, trim poor quality bases, and filter short reads. Quality filtered and primer trimmed FASTQ files were converted to FASTA [25] format and exported to the Quantitative Insights into Microbial Ecology (QIIME) software [26] after re-formatting read headers to be QIIME-compliant with a custom Perl script. 

### 2.5. Data Analysis

Reads were clustered into Operational Taxonomic Units (OTUs) based on 97% sequence similarity (similar to genus level) using default parameters within QIIME and a representative sequence for each OTU was chosen for downstream analysis based on the most abundant sequence from each OTU. Phylogenetic classification was assigned using the Ribosomal Database Project database (RDP) 22 release database within QIIME. Alpha and beta-diversity were calculated in QIIME after sequences were rarefied. Analysis was performed following standard procedures [27] at default settings for all scripts run and 132,600 reads per sample for beta diversity analysis. In order to determine richness and diversity, the Chao1 index [28] and Shannon index [29] were calculated. To confirm differences in the abundances of individual taxonomy between the two groups, Metastats software was utilized [30]. Additionally, the dissimilarity between the moderate exposure and low exposure groups (Beta diversity) was shown as a square matrix of “distance”. The data in this distance matrix were visualized with Principal Coordinate Analysis (PCoA) calculated with QIIME [27].

STATA software version 11.0 (StataCorp, College Station, TX, USA) was employed to analyze clinical characteristics. Mann-Whitney tests were used to compare age and arsenic concentration in drinking water and urine between groups. Simple linear regression analysis was applied to determine the relationship between arsenic in drinking water and the urine. Fisher’s exact test was performed to compare demographic characteristics and medical history between exposure groups. We also performed Fisher’s exact test to compare the prevalence of specific taxa between two groups with R software Version 3.01 [31] including the calculation of 95% confidence intervals for the odds ratios and the P values were adjusted for multiple comparisons. A *p* value of ≤ 0.05 was considered statistically significant in our study.

## 3. Results and Discussion

### 3.1. Results

The 20 sputum cell pellets used in this study were from subjects between 20 and 76 years old residing in Obregon, Mexico (Table 1). All subjects were Hispanic and current non-smokers, verified by personal questionnaire [21], and were predominantly female (85%). The self-reported medical history of participants is listed in Table 1. None were currently on medications. The mean concentration of arsenic in household water was 21.1 ppb for the moderate arsenic exposure group and 2.4 μg/ for the low arsenic exposure group. The sum of arsenic species in urine were significantly higher in the moderate than in the low exposure group (38.7 ppb and 19.5 ppb respectively, *p* < 0.01). Household arsenic concentration in drinking water was correlated (r^2^ = 0.42) with urinary arsenic concentration. Six subjects from the moderate exposure group were identified as having sum of arsenic species in their urine samples totaling above 35 ppb (μg/L), which is the American Conference of Governmental Industrial Hygienists (ACGIH) Biological Exposure Index [32].

After initial PGM filtering, 13.14 × 10^6^ sequences were obtained. There were a total of 3,920,441 sequence reads, ranging from 37,935 to 508,787 reads per sample for 316 (100 Mb) chips, generated after QIIME quality filtering. The number of sequences between groups was not significantly different (low exposure = 442,070, moderate exposure = 494,149, *p* = 0.41). We performed analysis using the lowest common denominator sample, with 37,935 reads, to control sample heterogeneity. Total operational taxonomic units (OTUs) at 97% sequence similarity ranged from 851 to 4,424 OTUs (Table 2). No significant difference in the number of OTUs between the case and control groups was observed (low exposure = 2,331.6, moderate exposure = 2,440.6, *p* = 0.24). In order to determine richness and diversity, Chao1 and Shannon Indices were calculated, with no significant differences resulting (*p* = 0.38 and *p* = 0.42 respectively).

**Table 1:** Demographic and physiological parameters.

	Moderate Exposure	Low Exposure	*p* Value
	(n or mean ± SD)	(mean ± SD)	
Number of Subjects	10	10	
Female	8	9	0.500
Age	51.0 ± 21.3	41.5 ± 13.2	0.122
Past smoking	4	1	0.151
Asthma	0	2	0.237
Chronic bronchitis or emphysema (COPD)	0	2	0.237
Diabetes	2	0	0.237
Cardiovascular disease	4	2	0.314
GI disease	1	2	0.500
Kidney disease	1	2	0.500
Liver disease	0	1	0.500
Neurological disease	2	1	0.500
As levels in water	21.1 ± 6.4	2.4 ± 0.8	<0.001
(μg/L, range) ^a^	(13.8–28.8)	(1.7–3.5)	
Sum of species (μg/L)^b^	38.7 ± 18.0	19.5 ± 7.0	<0.003

Notes: Cardiovascular disease includes heart disease, hypertension and peripheral vascular disease; ^a^ Arsenic levels in water (μg/L) from an unfiltered water source by household; ^b^ Sum of species in urine = As III + As V + MMA V + DMA V.

**Table 2:** Summary of sequence processing.

	Moderate Exposure	Low Exposure	*p* Value
	(mean ± SD)	(mean ± SD)	
Sequence Reads ^a^	494,149.4 ± 112,755.1	442,070.4 ± 102,308.5	0.368
OTUs	2,440.6 ± 357.3	2,331.6 ± 270.1	0.405
Chao1	3,491.1 ± 433.3	3,313.3 ± 342.5	0.376
Shannon Index	5.06 ± 0.25	5.00 ± 0.20	0.419

Note: ^a^ The read outputs are the outcome of QIIME qualified filtering.

**Figure 1:**
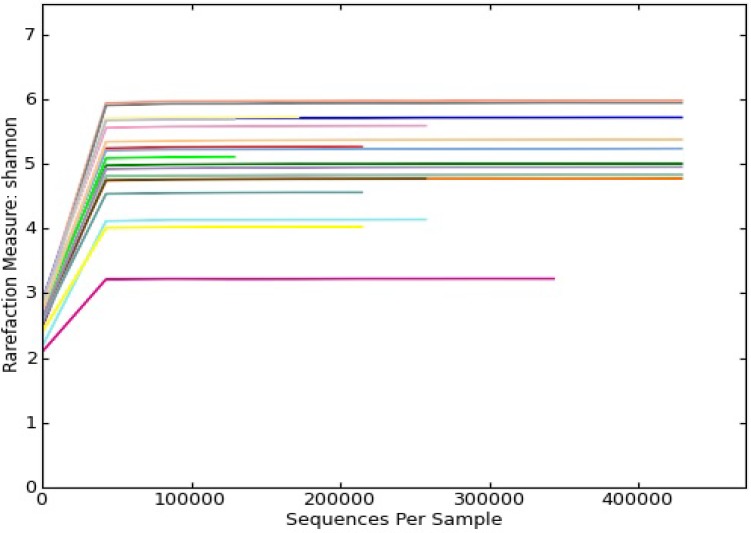
Shannon diversity collector curves.

**Figure 2:**
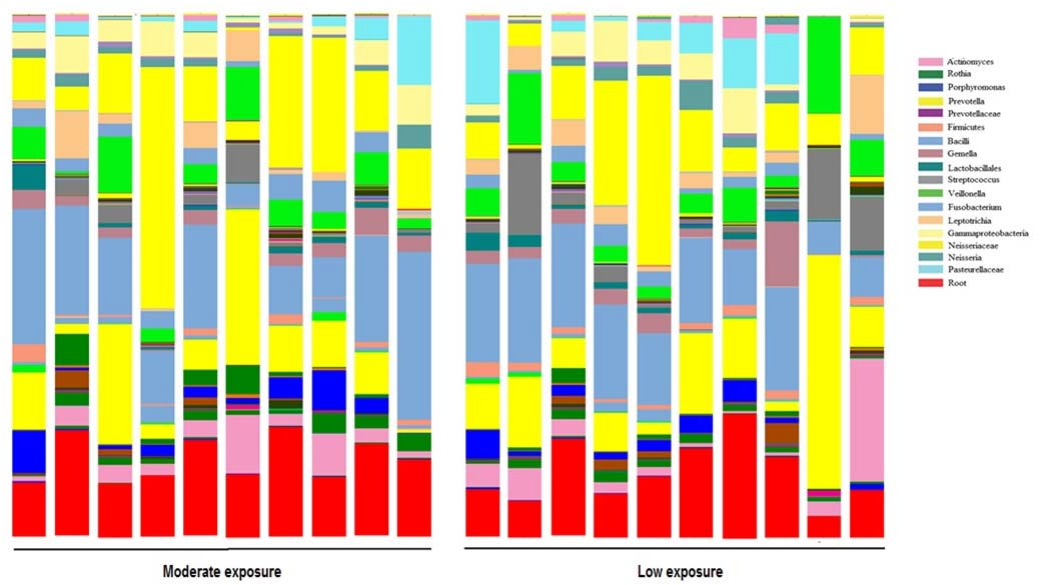
Bacterial community composition of sputum samples: Taxa assignments for each sample at the level of genus.

Taxa were determined by sequence similarity groupings (OTUs). Multiple rarefaction curves using the Shannon index values demonstrated that all curves reached saturated plateau phase (Figure 1). Our rarefraction analysis was done at the lowest value possible, namely 128,710 sequences per sample. Microbiomes for each of the subjects were characterized by phylogenetic analyses (Figure 2). Each bar represents microbiota analyzed from individual sputum samples. It is further partitioned into bacteria composition. The each color in the bar represents the each genus and only groups with >1% abundance are shown. The sequences unidentified to genus level were assigned to the lowest level of taxonomy identification. Poor phylogenic identification was categorized as bacterial root. Approximately 90% of sequences were classified at phylum level, with *Firmicutes* (22%), *Proteobacteria* (17%) and *Bacteriodetes* (12%) being the dominant phyla in all samples. At the family level, 85 individual families were identified, with 11 of them having a relative abundance of more than one percent in each sample. The three most common families in all samples were *Neisseriaceae* (15%), *Prevotellaceae* (12%) and *Veillonellacea* (7%). Of the 142 individual genera assigned 11 had a relative abundance of more than one percent, with *Prevotella* (11%), *Veillonealla (*6%) and *Actinomyces* (4%) commonly found.

The differentially abundant texa between groups were evaluated at the genus level using Metastats software [30] (Table 3). The taxonomic assignment was used for the lowest level of identification when it was not successfully classified at the genus level. Only the taxa with greater than 1% sequence abundance in at least one sample were analyzed, and none of these differences were statistically significant. Using Fisher’s exact test, the following taxa had a greater prevalence in the moderate exposure group than the low arsenic exposure group: *Firmicutes*, *Gemella*, *Lactobacillales, Streptococcus, Neisseria* and *Pasteurellaceae* within Fisher test 95% confidence level. Further, *Rothia*, *Prevotella*, *Prevotellaceae Fusobacterium* and *Neisseriaceae* occurred more frequently in the low arsenic exposure group. However, these differences were not statistically significant when analyzed using the Metastats q value.

**Table 3:** Differences in microbial community between moderate exposure and low exposure groups.

	Number of OTU		The Mean Proportion of OTUs in the Group (%)				
Taxon	Moderate exposure	Low exposure	Moderate exposure ^a^	Low exposure ^a^	*p*-value ^a^	Odds ratio ^b^	95% CI ^b^
*Actinobacteria /Actinomyces*	912	906	5.5%	5.0%	0.90	0.96	0.87–1.06
*Actinobacteria /Rothia*	326	415	1.6%	2.4%	0.15	0.75	0.64–0.87
*Bacteroidetes /Porphyromonas*	609	640	2.7%	3.7%	0.46	0.91	0.81–1.02
*Bacteroidetes /Prevotella*	2344	2497	14.4%	12.7%	0.80	0.89	0.83–0.94
*Bacteroidetse /Prevotellaceae*	202	484	0.9%	2.4%	0.19	0.39	0.33–0.47
*Firmicutes*	353	251	1.7%	1.3%	0.45	1.35	1.14–1.59
*Firmicutes /Bacilli*	3910	3884	19.1%	21.2%	0.62	0.95	0.91–1.00
*Firmicutes /Gemella*	712	552	4.0%	3.1%	0.76	1.24	1.11–1.39
*Firmicutes /Lactobacillales*	347	267	1.8%	1.4%	0.63	1.25	1.06–1.47
*Firmicutes /Streptococcus*	865	478	6.0%	2.3%	0.11	1.76	1.57–1.97
*Firmicutes /Veillonella*	1269	1217	8.0%	6.3%	0.52	1.00	0.92–1.08
*Fusobacteria /Fusobacterium*	540	713	2.6%	4.0%	0.11	0.72	0.64–0.80
*Fusobacteria /Leptotrichia*	810	744	4.1%	3.5%	0.74	1.04	0.94–1.15
*Proteobacteria /Gammaproteobacteria*	964	969	4.6%	5.3%	0.64	0.95	0.86–1.04
*Proteobacteria /Neisseria*	493	317	2.2%	1.8%	0.65	1.50	1.29–1.73
*Proteobacteria /Neisseriaceae*	2930	3190	14.6%	19.8%	0.43	0.86	0.82–0.91
*Proteobacteria/Pasteurellaceae*	1311	542	6.2%	3.5%	0.34	2.39	2.15–2.64

Note: ^a^ Metastats analysis performed to calculate mean levels of OTUs and *p*-value for the differential abundance between the two groups; ^b^ Odds ratios for presence of OTUs being present in the moderate exposure group as compared with the low arsenic exposure group for each taxon and 95% confidence intervals from Fisher’s exact test.

**Figure 3:**
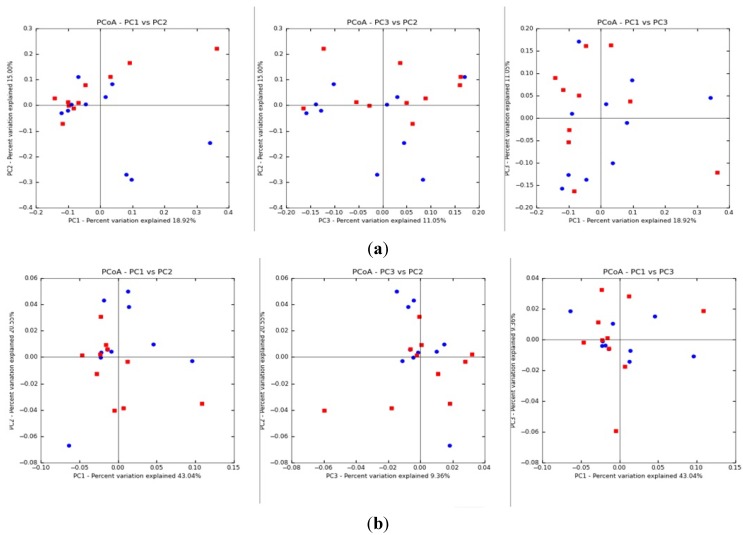
Unweighted (**a**) and weighted (**b**) beta diversity. Beta diversity comparison between exposure groups: each point indicates sputum samples from either moderate exposure (red) or low exposure (blue) groups.

Within the microbial community cluster, there appears to be no difference in patterns between the moderate exposure group and the low exposure group using the principal coordinate analysis (PCoA) of the UniFrac distance matrix (*p* = 0.18) (Figure 3). 

### 3.2. Discussion

Our study investigated, for the first time, the microbiota of sputum from subjects exposed to moderate or low arsenic concentrations through drinking water. Prior studies have demonstrated that diverse microbial populations exist in the lungs of healthy humans [11,13,14]. Utilizing high-throughput sequencing of 16s RNA, *Proteobacteria*, *Firmicutes*, and *Bacteroidetes* have been most commonly identified at the phylum level. At the genus level, *Pseudomonas*, *Streptococcus*, *Prevotella*, *Fusobacteria*, and *Veillonella* predominate, with lesser contributions from potential pathogens, including *Haemophilus* and *Neisseria* [33]. Our study found that the most core bacteria among subjects were *Firmicutes Firmicutes* (22%), *Proteobacteria* (17%) and *Bacteriodetes* (12%) at the phylum level, which were similar patterns reported for the upper respiratory tract in healthy subjects [33]. However, we found several rare bacteria in the upper respiratory tract, including *Gemella* and *Leptotrichia*, generally found in oral flora [34]. We asked subjects to spit out saliva while collecting sputum samples to prevent contamination from the oral microbiome but this procedure likely does not exclude all contamination. 

While the authors are unaware of any prior human microbiota studies elucidating the effect of arsenic exposure from drinking water, published data have demonstrated impact of arsenic upon the microbiota in soil. In a recent study, the bacterial population in soil contaminated by chromium and arsenic showed reduced diversity and shifted phylogeny [35]; the diversity of the microbial community in the arsenic contaminated soil declined by 14% for species richness, evenness, and diversity indices compared to non-arsenic contaminated soils. Also, a significant shift in dominance from *Actinobacteria* in control soils to *Proteobacteria* in arsenic samples indicates that *Proteobacteria* may be the most metal tolerant organisms found at arsenic contaminated sites [35]. 

In our study, there was an increase in *Streptococcus* in the moderate arsenic exposure group compared to the low exposure group. A novel integrative conjugative element (ICE), ICESde3396, from *Streptococcus dysgalactiae* subsp*. equisimilis* (group G streptococcus [GGS]), a bacterium commonly found in the throat and skin of humans, was reported in a recent study, and it carries genes involved in an increased resistance to various metals, such as cadmium and arsenic [36]. This trait could potentially affect the prevalence of *Streptococcus* in the respiratory tract of exposed individuals. 

Prior to culture independent methods, approximately 1% of microbial diversity in given sample was assessed in bacterial species in a given environment [37]. However, the development of culture-independent methods and the commercialization of next-generation sequencing technology have resulted in novel bacterial phyla detection, including uncultivable organisms [38]. The key advantages of the Ion PGM Platform sequencing used in our study are economy and speed, due to the use of unmodified nucleotides [39]. This technology has been used to measure microbial diversity in a environmental samples [40] and sub gingival plaque [41]. In conjunction with next-generation technology, 16S rRNA sequencing provides more accurate estimation of species richness and accessibility of rare bacterial communities [42]. Since 1,550 base pair long (relatively small) 16Sr RNA possess both a highly conserved and variable locus of the bacterial genomes, its analysis becomes a standard in understanding bacterial species and performing taxonomic studies [43]. For the 16S rRNA gene amplicon sequencing, we targeted the V6 hypervariable region, corresponding to positions 986–1054 *(Escherichia coli*). In spite of the short length of the region, it has successfully identified various types of phylotypes with convenience [37]. Though this method is criticized as having low efficacy [40], it is still valuable for community comparisons of species richness and diversity [41]. In our study, about 39% of sequences remained unidentified at the genus level.

Limitations of our study include a small sample size and relatively moderate arsenic exposures. Based on our study results, we estimate that our sample size of 10 in each exposure group provided us 70% power to detect an OTU effect size of 1.1, with an adjusted false discovery rate of 0.05. Other population groups have had considerably greater exposures, for example in Bangladesh where groundwater contamination ranged up to 910 ppb of inorganic arsenic [44]. In animal studies, even concentrations of arsenic below the EPA MCL of 10 ppb altered the signaling pathway and innate immunities in the lung [9,45]. A cohort study showed a higher prevalence of adverse respiratory symptoms such as chronic cough, breathing problems and blood in sputum with exposures over 7 ppb of arsenic, compared to exposures below 7 ppb [46]. While our subjects were exposed to relatively moderate and low levels of arsenic, in most studies of subjects with clinical diseases, including non-malignant lung disease, arsenic exposures were much higher [8,47]. Also, our study subjects included ex-smokers and individuals with self-reported respiratory disease, and we did not have objective assessments of outcome status, such as medical records or physical exam findings, all of which may have influenced the microbiome. A larger study will be necessary to confirm the results of our exploratory study. 

The current study also had a disproportionate number of women. Prior studies have described the possibility of sex differences in human arsenic metabolism [48,49]. A study of a population of West Bengal, India, exposed to arsenic via drinking water reported that strong pulmonary effects were found among the male population, but not in females [5]. It is possible that the female dominant population in the present study potentially influenced the results observed. It has also been suggested that females may be less susceptible to arsenic toxicity due to the much greater frequency of skin lesions in males than in females (odds ratio 10.9 *vs.* 5.78, *p* = 0.005) [50]. 

Other studies have been conducted to explore different features of microbiome by geography and individual [51,52]. It has been shown that features of microbiomes on the digestive tracts are unique to different locations and lifestyles. The uniqueness of each individual’s microbial community depends on the site that was stressed [53]. Oral and stool communities are especially diverse compared to simpler bacterial communities such as vaginal sites among individual [53]. Variability in individual pulmonary microbiota likely influenced our ability to detect significant differences in microbiome diversity between the moderate and low exposure arsenic groups, and given our small sample size, may have also resulted in extraneous findings as well. 

## 4. Conclusions

In conclusion, in our exploratory study we did not find significant effects of moderate arsenic exposure on measures of overall respiratory microbiota diversity, although differences between moderate and low arsenic exposure groups in specific genera were noted. Additional research with more subjects exposed to a greater range of arsenic concentrations is needed to confirm our study findings and further characterize the effects of arsenic on lung microbiota. 
